# Amaranth (*Amaranthus caudatus* L.): Nutritional Composition, Bioactive Compounds, Processing Technologies, Food Applications, and Future Perspectives

**DOI:** 10.3390/molecules31172960

**Published:** 2026-08-25

**Authors:** Katherine Alva-De-La-Cruz, Gian Pierre Silvera-Otañe, Cesar Moreno-Rojo, Marcio Schmiele, Luz María Paucar-Menacho

**Affiliations:** 1 Programa de Doctorado en Ingeniería Agroindustrial, Mención Transformación Avanzada de Granos y Tubérculos Andinos, Universidad Nacional del Santa, Nuevo Chimbote 02712, Peru; 2025818015@uns.edu.pe (K.A.-D.-L.-C.); 2025818011@uns.edu.pe (G.P.S.-O.); 2 Departamento Académico de Agroindustria y Agronomía, Facultad de Ingeniería, Universidad Nacional del Santa, Nuevo Chimbote 02712, Peru; cmoreno@uns.edu.pe; 3 Laboratório Integrado de Cereais e Lipídeos (LICEL), Instituto de Ciência e Tecnologia (ICT), Universidade Federal dos Vales do Jequitinhonha e Mucuri (UFVJM), Diamantina 39100-000, Brazil; marcio.sc@ict.ufvjm.edu.br

**Keywords:** Andean grains, phenolic compounds, antioxidant activity, peptides, gluten-free foods, functional ingredients, sustainable nutrition

## Abstract

Amaranth (*Amaranthus caudatus* L.) is an Andean pseudocereal with a nutritional profile characterized by its protein, dietary fiber, and bioactive compound content, adequate balance of essential amino acids, and the presence of bioactive compounds with antioxidant, anti-inflammatory, and cardiometabolic properties. This review analyzes the main processing methods applied to amaranth, including milling, roasting, popping, rolling, extrusion, germination, fermentation, encapsulation, and component isolation, describing how these technologies modify its nutritional, functional, and technological properties. It also examines its applications in various food categories, such as baked goods, extruded snacks, fermented beverages, symbiotic foods, and gluten-free formulations, highlighting its potential as a functional food ingredient for developing functional foods and nutraceuticals. Furthermore, this study discusses the technological and industrial challenges associated with its processing, including structural, sensory, and scalability limitations, within the context of the growing global demand for healthy and sustainable foods. Finally, the study identifies knowledge gaps and research priorities to optimize processing conditions, improve sensory acceptability, and enhance industrial value, thereby supporting further evaluation of its integration into food production systems.

## 1. Introduction

Andean grains, primarily represented by amaranth (*Amaranthus caudatus* L.), quinoa (*Chenopodium quinoa*), and kañiwa (*Chenopodium pallidicaule*), have gained increasing international attention due to their nutritional quality and remarkable ability to withstand adverse environmental conditions [1,2]. These crops are internationally recognized as “superfoods” or “seeds of the 21st century” due to their high content of high-quality protein, adequate balance of essential amino acids, and richness in fiber, minerals, and bioactive compounds [3,4,5,6]. Furthermore, their high resilience to adverse climatic conditions, such as drought and salinity, positions them as strategic resources for ensuring global food security despite the challenges of climate change [3,7,8,9].

In this context, the food industry is facing a growing demand for products that, in addition to providing nutritional value, offer preventive health benefits, driving the development of functional foods and nutraceuticals [10,11,12]. This trend aligns with the protein transition, in which consumers seek to replace animal-based proteins with more sustainable and nutritionally balanced plant-based alternatives [12,13]. At the same time, the increasing prevalence of chronic-related diseases (CRDs) and the preference for clean-label products have fueled the boom in gluten-free foods, positioning pseudocereals as key ingredients in the development of baked goods, snacks, and beverages [9,14,15,16]. In this context, priority is given to processing technologies that preserve the stability of bioactive compounds and reduce the use of artificial additives [9,10,12].

**Figure 1:**
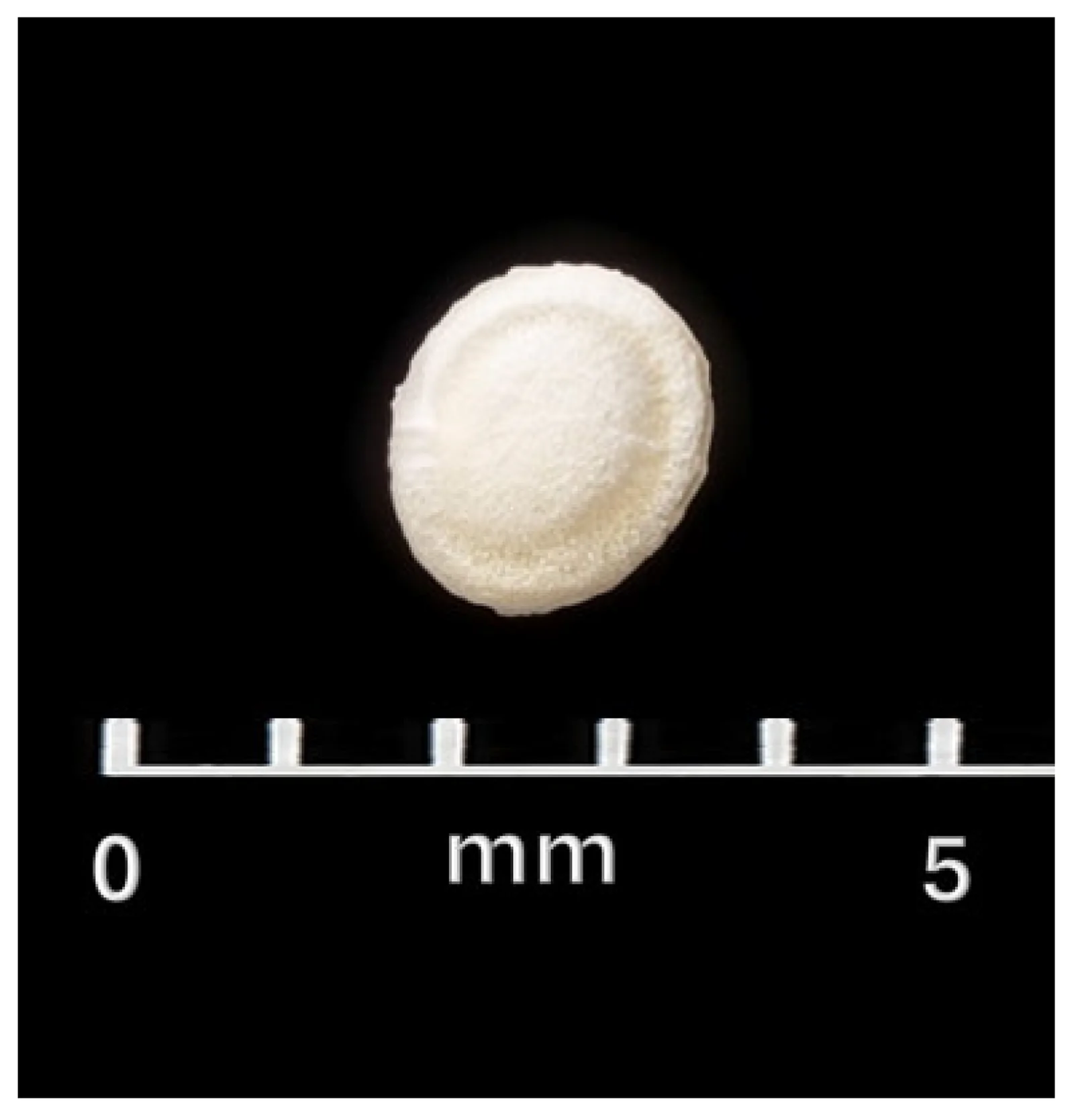
Close-up view of a single amaranth seed showing its physical appearance and size, with a reference scale in millimeters.

In this regard, amaranth is recognized as one of the most nutritionally valuable Andean pseudocereals due to its nutritional composition and protein quality compared with several conventional cereals. The small size of amaranth seeds is a distinctive physical characteristic, with individual grains typically measuring only a few millimeters in diameter, as illustrated in Figure 1. Its protein content may reach 16%, with a balanced essential amino acid composition and particularly high levels of lysine, a nutritionally limiting amino acid in wheat, rice, and corn [11,16,17]. The nutritional relevance of amaranth extends beyond its macronutrient composition and is strongly linked to its bioactive compounds and their reported biological activities, including antioxidant, anti-inflammatory, hypocholesterolemic, and antidiabetic properties, which are primarily attributed to the presence of bioactive compounds such as squalene, polyphenols, and bioactive peptides [18,19]. These compounds have been associated with the modulation of oxidative stress [18], inflammatory pathways [18], lipid metabolism [7,16], and glycemic regulation [7]; however, the evidence supporting these effects varies according to the compound, experimental model, and study design [3,19]. Furthermore, from a technological and industrial perspective, amaranth exhibits potential for incorporation into a wide range of value-added products, including fortified flours, extruded snacks, breakfast cereals, energy bars, pasta [12,15], fermented foods, and malted beverages [4]. In addition to its nutritional and functional advantages, the crop demonstrates high adaptability to marginal environments and requires relatively low water inputs, which may contribute to the development of more resilient agri-food systems under climate change scenarios [9,16].

Despite its nutritional and functional potential, the broader utilization of amaranth remains constrained by limited industrial standardization, variable raw material characteristics, and insufficient integration of processing strategies with product development [3]. These limitations highlight the need for further research to support its consistent utilization and commercialization in functional and value-added foods [9,12]. The available evidence is fragmented, and there is limited integration between processing technologies and their effects on the grain’s bioactivity, bioavailability, and technological performance, in addition to low standardization of experimental conditions. Likewise, systematic information on certain processes, such as germination, fermentation, and extrusion, remains limited compared to pseudocereals like quinoa, and there is still little knowledge about the nutritional potential of specific ecotypes, such as black and pink grains, which remain underutilized in the Andean region. Moreover, the health benefits associated with amaranth remain largely supported by in vitro and preclinical studies, highlighting the need for well-designed clinical trials to substantiate its functional claims [1,10]. Additionally, greater attention should be devoted to genotype–environment interactions, as these factors play a crucial role in determining the synthesis, accumulation, and stability of bioactive compounds, ultimately influencing the nutritional and nutraceutical value of the grain. In this context, an integrated approach is needed to understand the interactions between processing, functionality, and health [7,11]. Therefore, this review aims to: (i) summarize current information on its nutritional composition, highlighting its nutritional profile and the presence of bioactive compounds, such as peptides, squalene, and phenolic compounds; (ii) evaluate the main conventional technological processes, such as milling, roasting, puffing, rolling, and germination, as well as traditional biotechnological processes with emerging applications, such as fermentation, and emerging technologies, such as extrusion, encapsulation, enzymatic hydrolysis, and component isolation, and their influence on technofunctional properties and nutrient availability; (iii) analyze its applications in various food products, ranging from baked goods, extruded snacks, and breakfast cereals to fermented beverages and meat analogs; and (iv) examine the main challenges and opportunities for its sustainable use, considering food safety aspects, the need for clinical validation, and its relevance in the face of climate change. Overall, this review seeks to integrate current evidence on amaranth composition, processing, functionality, and food applications, while identifying relevant research gaps and technological challenges.

## 2. Methodology

The present narrative review was developed through a structured literature search conducted in major scientific databases, including Scopus, ScienceDirect, and SciELO. Studies published between 2021 and January 2026 were considered, with emphasis placed on the most recent and scientifically relevant evidence. The search strategy combined the terms “amaranth” and “*Amaranthus caudatus*” with keywords related to food applications, bioactive compounds, phenolic compounds, nutritional properties, and processing technologies. Additional search terms included “germination”, “fermentation”, “extrusion”, “milling”, “encapsulation”, “protein isolate”, “bread”, “cookies”, “flour”, “bakery products”, “beverages”, “snacks”, and “functional ingredients”. The selection process prioritized peer-reviewed research articles and review papers available in full text, published in English or Spanish, and indexed in high-impact scientific databases. Studies published before 2021, studies lacking full-text availability, conference abstracts, duplicated records, and articles that were not directly relevant to the scope and objectives of this review were excluded. Studies addressing the food and technological applications of amaranth were specifically considered, as these topics constitute important components of the present review.

This manuscript is a narrative review that synthesizes the available evidence on the nutritional composition, bioactive compounds, processing technologies, digestibility, bioavailability, and food applications of amaranth.

## 3. Proximal Composition

The proximate composition of amaranth seeds highlights their nutritional value and varies according to genotype, geographical origin, environmental and agronomic conditions, maturity stage, postharvest handling, and analytical methodology [11,16,20,21]. Amaranth is an important source of protein, dietary fiber, minerals, and lipids, with its mineral fraction containing calcium, iron, phosphorus, magnesium, and zinc, while its lipid fraction is composed predominantly of unsaturated fatty acids [14,22]. The grain also contains bioactive compounds such as squalene, phytosterols, tocopherols, and phenolics [10,17], which have been associated with antioxidant and other biological activities [11,22].

Furthermore, the absence of gluten proteins makes amaranth particularly suitable for individuals affected by celiac disease, non-celiac gluten sensitivity, gluten ataxia, and wheat allergy. Together with its nutritional composition and adaptability to adverse environmental conditions, these characteristics support its potential contribution to dietary diversification and sustainable food systems [14,23,24].

Table 1 summarizes the reported proximate composition of amaranth in comparison with other pseudocereals and cereals. The values reported in the literature show considerable variability, which should be considered when interpreting comparisons among different grains, particularly because differences in genotype, cultivar, growing conditions, processing state, and analytical methodology may influence the reported composition.

### 3.1. Proteins

The protein content in amaranth seeds ranges between 12 and 16% [2,23], and is distinguished by a well-balanced essential amino acid (EAA) profile, particularly its high lysine content [11,21]. Among the non-essential amino acids (NEAs), glutamic acid predominates, reaching concentrations up to 24.8 g·100 g^−1^ of protein, followed by aspartic acid and arginine, the latter playing important roles in child growth, immune function, and metabolic regulation [7]. Nevertheless, variability in amino acid composition among ecotypes and cultivation environments indicates that these values are not constant and should be interpreted considering genetic and environmental influences [24,25].

The nutritional quality of amaranth proteins is associated with their protein fraction composition, which is dominated by albumins (~40%) and globulins (~20%). These fractions contribute to a favorable essential amino acid profile, particularly the relatively high lysine content, and therefore help explain the high biological value of amaranth proteins compared with conventional cereal proteins [25]. Furthermore, bioprocessing strategies such as controlled or elicited germination can further enhance these results by inducing de novo protein synthesis, promoting reserve mobilization and metabolic activation, resulting in increases of up to 17.9% in total protein in black-grain ecotypes [11].

From an industrial perspective, these characteristics reinforce the potential of amaranth as a valuable ingredient for functional and gluten-free foods [14]. Beyond serving as a source of high-quality plant protein, amaranth protein hydrolysates can release bioactive peptides with antihypertensive and antioxidant activities, contributing to improved metabolic health [21,23,24]. Furthermore, previous studies have reported a chemical score exceeding 100 and in vitro protein digestibility values ranging between 80 and 96% following extrusion, outperforming conventional cereals such as whole yellow corn and polished white rice [2]. These findings indicate that amaranth proteins may provide favorable nutritional characteristics compared with some conventional cereal proteins, although protein quality and digestibility depend on processing conditions and product formulation [15,17].

### 3.2. Lipids

The lipid content in amaranth seeds typically ranges from 2 to 14%, depending on the ecotype and cultivation conditions [2,11]. Its lipid fraction is predominantly composed of unsaturated fatty acids (75–77%), with linoleic acid (ω-6) representing the major fatty acid (41–62%). Amaranth is also recognized as an important plant source of squalene (2.4–12% of the oil). The broad variation in lipid composition reported across studies reflects differences in genotype, environmental conditions, and agronomic practices, highlighting the influence of both genetic and environmental factors on lipid biosynthesis [21,23].

The predominance of unsaturated fatty acids appears to be closely associated with the adaptation of amaranth to the harsh environmental conditions of the Andean highlands. The accumulation of polyunsaturated lipids helps preserve membrane fluidity and maintain electron transport efficiency under low-temperature stress. At the molecular level, this response has been linked to increased expression of FAD2 desaturase genes, which promote fatty acid desaturation while limiting lipoxygenase-mediated pathways that are more commonly observed in tropical species [26].

Beyond its nutritional importance, the lipid fraction of amaranth has attracted considerable interest for nutraceutical applications [27]. Squalene, tocopherols, and phytosterols have been associated with cardioprotective effects through complementary mechanisms, including modulation of cholesterol metabolism via inhibition of HMG-CoA reductase, resulting in reductions in LDL cholesterol and circulating triglycerides in experimental models. Nevertheless, evidence from human intervention studies remains scarce, and the magnitude of these effects is likely influenced by factors such as dosage, food matrix, and the bioavailability of these bioactive compounds [10,18].

Compared with conventional cereals and other cultivated amaranth species, including *Amaranthus cruentus* L. and *Amaranthus hypochondriacus* L., amaranth generally exhibits a higher concentration of linoleic acid and squalene [26]. This lipid profile has contributed to interest in amaranth as a plant-based source of squalene for food and nutraceutical applications [18,25,28].

### 3.3. Available Carbohydrates

Carbohydrates represent the major macronutrient in amaranth grains, accounting for approximately 55–66% of their dry weight [2,21,23]. The carbohydrate fraction is predominantly composed of waxy starch, characterized by a low amylose content (5–7%) and a correspondingly high proportion of amylopectin [29]. Nevertheless, considerable variation in starch composition has been reported among ecotypes and cultivation environments, which partly explains the differences observed in the technological and functional properties of amaranth-based products [22,28].

The distinctive functional behavior of amaranth starch is closely related to its unique granular structure. The starch granules are extremely small (0.3–3 µm) and predominantly polygonal, providing a large specific surface area that facilitates water absorption and enzymatic hydrolysis [21,28,30]. In addition, non-starch polysaccharides, such as pectins and xyloglucans, contribute to high swelling capacity and water retention, improving the rheological performance of the flour during thermal processing [22,25].

These structural characteristics make amaranth particularly attractive for the development of gluten-free and expanded food products. The low amylose content of amaranth starch contributes to its gelatinization behavior and influences the development of the starch matrix during thermal processing. However, expansion during extrusion is a multifactorial phenomenon governed by the combined effects of the amylose–amylopectin ratio, starch gelatinization, moisture content, melt viscosity, protein and fiber composition, screw speed, temperature, and die pressure. Therefore, the low amylose content should be considered one contributing factor rather than a direct determinant of expansion. Although amaranth contains less total carbohydrate than conventional cereals such as polished white rice (80%) and whole yellow corn (74%), its starch exhibits superior digestibility and technological functionality, especially in gluten-free formulations [21]. Previous studies have also demonstrated that the microstructure of amaranth starch improves paste stability and processing performance when compared with other pseudocereals, reinforcing its value as a functional food ingredient [8,29].

### 3.4. Dietary Fiber

Amaranth is an excellent source of dietary fiber, with reported content ranging between 2.7 and 17.3% depending on the genotype and cultivation conditions, while insoluble fiber accounts for approximately 78–80% of the total fiber fraction [2,7,25]. This composition is primarily attributed to the abundance of structured polysaccharides, including arabinose-rich pectins (34–55%) and xyloglucans (40%), which constitute the architecture of the grain cell wall. Processing technologies, such as germination, can further modify this fraction through enzymatic remodeling of cell wall components and mobilization of reserve compounds, although the extent of these changes depends strongly on processing conditions [15,31].

The nutritional relevance of amaranth fiber extends beyond its quantitative content. Its consumption has been associated with a low-to-moderate glycemic index (GI ≈ 47.65), prebiotic activity, and improved cholesterol metabolism, supporting its role in the prevention of chronic non-communicable diseases [25]. From a technological standpoint, the high water-holding capacity of the fiber fraction contributes to improved dough rheology, moisture retention, and texture development in products such as pasta, bakery products, and energy bars [15,23,32].

Compared with refined cereals, amaranth provides substantially higher amounts of dietary fiber content. For example, refined rice contains approximately 0.1% fiber, whereas corn contains about 2.7%, values considerably lower than those reported for most amaranth ecotypes. Among them, the Centenario variety has been highlighted for its particularly high fiber concentration, reinforcing the potential of amaranth as a functional ingredient for gluten-free foods and for dietary strategies aimed at improving gastrointestinal health and reducing the risk of chronic diseases, including those affecting individuals with celiac disease [25,27].

### 3.5. Ash

The ash content in amaranth seeds generally ranges from 2 to 3.8%, although higher values have been reported following bioprocessing treatments such as controlled germination [11,21]. For example, germination for 72 h may increase the relative ash content by up to 18.8% on a dry weight basis, which is attributed to a concentration effect resulting from the loss of organic dry matter (carbon dioxide and water) during seed respiratory metabolism [7,11,21]. This increase is primarily attributed to the progressive loss of dry matter through respiration and enzymatic degradation of reserve compounds, particularly carbohydrates and lipids, which results in a relative concentration of the mineral fraction. In addition, mineral remobilization toward developing tissues during germination contributes to this response, as several minerals serve as essential cofactors for enzymes involved in early seedling metabolism [1,11,31].

Mineral composition also varies among amaranth ecotypes. Black-seeded varieties generally exhibit higher ash and mineral contents than pink-seeded ecotypes, reflecting genetic differences and physiological adaptations that influence nutrient uptake and accumulation under Andean growing conditions [11,33].

The relatively high ash content of amaranth highlights its importance as a valuable source of essential minerals, particularly calcium, iron, and magnesium. Representative concentrations of calcium, iron, and zinc in *Amaranthus caudatus* seeds range from 63.9 to 285 mg/100 g, 5.78 to 17.4 mg/100 g, and 1.07 to 5.90 mg/100 g, respectively, on a dry weight basis. These mineral concentrations may vary according to genotype and edaphoclimatic conditions, resulting in differences in mineral accumulation among amaranth ecotypes [27,33].

The relatively high ash content of amaranth highlights its importance as a valuable source of essential minerals, particularly calcium, iron, and magnesium. Consequently, the grain represents a promising ingredient for the development of nutrient-dense functional foods and gluten-free products aimed at improving mineral intake and reducing the risk of nutritional deficiencies [11,25,34].

When compared with conventional cereals, amaranth exhibits a markedly higher mineral content. Ash values reported for refined rice (approximately 0.4%) and wheat (around 1.76%) are substantially lower than those typically found in amaranth, supporting its recognition as one of the most mineral-rich pseudocereals. Nevertheless, these comparisons should be interpreted cautiously, since mineral composition may be influenced by cultivar, environmental conditions, grain refinement, and differences in analytical methodologies [8,15,21].

## 4. Bioactive Compounds, Digestibility, and Bioavailability

### 4.1. Bioactive Compounds in Amaranth

Phytochemical analysis demonstrates that amaranth possesses a complex and diverse bioactive profile (Table 2), encompassing phenolic compounds and betalains. The reported concentrations vary according to genotype, ecotype, cultivation environment, processing conditions, extraction procedures, analytical methods, product formulation, and reporting basis. For example, total phenolic content (TPC) in raw *Amaranthus caudatus* has been reported at 241 ± 1.84 mg GAE·100 g^−1^ dry weight in a black-seeded ecotype and 168 ± 1.81 mg GAE·100 g^−1^ dry weight in a pink-seeded ecotype. In the black ecotype, TPC increased progressively from 241 ± 1.84 mg GAE·100 g^−1^ in the raw grain to 655 ± 1.77, 869 ± 1.59, and 926 ± 1.58 mg GAE·100 g^−1^ after 24, 48, and 72 h of germination, respectively [11,28]. Similarly, total betalain content increased during germination, reaching 1.57 ± 0.04 mg BT·100 g^−1^ dry weight after 72 h [11]. Nevertheless, substantial variability in the phytochemical profile has been reported among studies, largely reflecting differences in genotype, cultivation environment, and processing conditions, extraction procedures, analytical methods, and reporting basis [4,11,30].

In addition to low-molecular-weight phytochemicals, amaranth is an important source of bioactive peptides generated through the hydrolysis of storage proteins. Some low-molecular-weight peptides, including fractions below 3 kDa, have shown inhibitory activity against angiotensin-converting enzyme (ACE) and dipeptidyl peptidase-4 (DPP-IV), suggesting potential antihypertensive and antidiabetic effects [35,36]. The accumulation and release of bioactive compounds may be enhanced during germination through the activation of endogenous enzymes and metabolic pathways. In particular, activation of phenylalanine ammonia-lyase (PAL) and the phenylpropanoid pathway can promote the synthesis and release of phenolic metabolites such as caffeic acid and rutin [11,37,38].

However, increases in the reported concentration of bioactive compounds during germination should be interpreted cautiously. Part of the apparent increase expressed on a dry weight basis may result from dry matter losses associated with respiration and the mobilization of carbohydrate and lipid reserves, as well as changes in compound extractability. Therefore, increased concentration should not necessarily be interpreted as equivalent to net biosynthesis or accumulation of the corresponding compounds.

Other bioactive constituents, such as squalene, have distinct physiological roles within the grain. Squalene, a linear triterpene, is considered part of the plant defense system and may contribute to membrane stability under environmental stress. In contrast, bioactive peptides may be released from storage proteins, particularly albumins and globulins, through endogenous proteolysis during germination and gastrointestinal digestion [11,27,36]. Together, these mechanisms may contribute to the functional properties associated with amaranth and its processed products.

The diversity of reported bioactive constituents has supported research into the use of amaranth and its derived ingredients in functional food and nutraceutical applications. Phenolic compounds, betalains, GABA, squalene, tocopherols, phytosterols, and bioactive peptides have been associated with antioxidant, hypocholesterolemic, antihypertensive, and antidiabetic activities, although the magnitude of these effects depends on factors such as bioavailability, food matrix, processing conditions, and experimental model [11,17,38]. In particular, plant-derived squalene has attracted growing interest as a sustainable alternative to conventional animal-derived sources [38,39].

Overall, the data compiled in Table 2 highlight the substantial variability in the reported bioactive composition of amaranth. Consequently, direct comparisons among studies should be made cautiously, particularly when different extraction procedures, analytical methods, sample matrices, or reporting units are used. These methodological and matrix-related differences may partly explain the wide variability reported for phenolic compounds and other bioactive constituents.

### 4.2. Antinutrients

Amaranth contains relatively low concentrations of antinutritional compounds, with phytic acid (InsP_6_) ranging from 2.9 to 7.9 g·kg^−1^ and tannins from 0.04 to 0.28 g·kg^−1^ [27,30,36]. Among these compounds, phytic acid represents the principal antinutrient (typically ranging from 2.90 to 7.90 g/kg dry weight in amaranth seeds) because of its strong ability to chelate essential minerals, including Ca^2+^, Fe^2+^, Fe^3+^, and Zn^2+^, forming insoluble complexes that reduce their intestinal absorption [27,33,40].

Although antinutrients reduce nutrient bioavailability, their concentration can be substantially decreased through appropriate processing technologies. Germination and fermentation are particularly effective because they activate endogenous phytases, promoting phytic acid degradation by up to 74% and consequently improving mineral bioavailability [30,41].

Compared with several cereal and pseudocereal species, amaranth exhibits a relatively favorable antinutritional profile. Phytic acid levels are generally lower than those reported for buckwheat and quinoa, and unlike quinoa, amaranth is naturally free of the high concentrations of bitter saponins that require extensive post-harvest removal. Moreover, phytate-related mineral bioavailability is better interpreted using phytate-to-mineral molar ratios than by phytate concentration alone. In particular, phytate-to-mineral molar ratios provide a more nutritionally meaningful estimate of the potential inhibitory effect of phytate on mineral absorption [7,15]. However, these ratios should only be calculated when phytate and mineral concentrations are reported for the same sample and under comparable analytical conditions; combining values from independent studies could produce misleading estimates. Therefore, the available evidence suggests that amaranth may exhibit a favorable phytate-to-mineral profile compared with some conventional cereals, particularly when appropriate processing strategies are applied to reduce phytate levels [7,30].

### 4.3. Digestibility and Bioavailability of Amaranth

Amaranth exhibits high nutritional quality owing to its favorable digestibility and bioavailability of essential nutrients. In vitro protein digestibility ranges from 70 to 80% in raw whole grains and can exceed 90% after extrusion or the production of instant porridge [2,21,36]. Despite its rich mineral composition, the bioavailability of calcium (73.60 mg·100 g^−1^) and iron (5.78 mg·100 g^−1^) is initially limited by antinutritional compounds, primarily phytic acid and oxalates, which reduce mineral absorption through chelation [27,36,40].

The excellent digestibility of amaranth carbohydrates is closely associated with the physicochemical characteristics of its starch. Its small, polygonal starch granules and waxy structure, characterized by low amylose content, facilitate rapid enzymatic hydrolysis by *α*-amylases, resulting in greater starch digestibility than that observed in many conventional cereals [17,29,31]. Protein digestibility also improves considerably after biological processing. Germination and fermentation activate endogenous phytases and proteases that simultaneously degrade phytic acid and hydrolyze storage proteins, increasing amino acid availability while enhancing mineral absorption [1,30]. Although these technologies consistently improve nutritional quality, the magnitude of their effects depends on processing conditions, genotype, and environmental factors, which partly explains the variability reported in the literature [7,27,30].

The combination of high digestibility, superior protein quality, and naturally low prolamin content makes amaranth particularly attractive for the development of gluten-free functional foods. These characteristics are especially relevant for individuals with celiac disease and for nutritional interventions aimed at reducing protein–energy malnutrition and micronutrient deficiencies [14,20,42].

Compared with conventional cereals such as wheat and corn, amaranth generally exhibits favorable protein digestibility and nutritional quality. Previous studies have reported biological values (BV) ranging from 70 to 80% and protein efficiency ratios (PER) between 1.39 and 4.81, indicating substantial variability among studies [5,14,21]. This variability suggests that protein quality is not a fixed characteristic of amaranth but depends on both biological and technological factors. Genotype and ecotype can modify amino acid composition and protein fraction distribution, whereas germination, fermentation, extrusion, and thermal processing may alter protein digestibility, amino acid availability, and the formation of protein–phenolic or protein–carbohydrate interactions. Differences in experimental models, protein sources, processing severity, and dietary formulations may further contribute to the variability reported among studies. Consequently, BV and PER values should be interpreted in relation to the specific genotype, processing history, and experimental conditions rather than as universal values for amaranth [14,27,36].

## 5. Nutraceutical Value and Functional Properties

### 5.1. Antioxidant Activity

Amaranth exhibits remarkable antioxidant potential, with ORAC values ranging from 18.55 to 114.92 µmol TE·g^−1^ and DPPH radical scavenging activity reaching 77.91% in protein hydrolysates [24,31,43]. Considerable variation has been reported among genotypes and processing conditions, with black-seeded ecotypes germinated for 72 h presenting the highest concentrations of total phenolic compounds (926 mg GAE·100 g^−1^) and antioxidant capacity (450 µmol TE·100 g^−1^), substantially exceeding the values observed for pink- and white-seeded varieties [11]. However, the apparent increase in phenolic concentration during germination should be interpreted cautiously. When expressed on a dry weight basis, part of the increase may result from a concentration effect associated with dry matter losses caused by respiration and the mobilization of carbohydrate and lipid reserves. Therefore, the increase in phenolic concentration cannot necessarily be attributed exclusively to de novo biosynthesis. Changes in absolute compound content, dry matter balance, and extraction yield should be considered when assessing the net effect of germination on phenolic accumulation.

The antioxidant activity of amaranth is primarily associated with the accumulation of phenolic compounds and other secondary metabolites during seed development and bioprocessing [9,37]. Germination plays a particularly important role by activating phenylalanine ammonia-lyase (PAL) and stimulating the phenylpropanoid pathway, thereby promoting the synthesis of antioxidant molecules such as caffeic acid and rutin. In addition, enzymatic hydrolysis and controlled thermal treatments facilitate the release of phenolic compounds previously bound to the cell wall, increasing their extractability and antioxidant activity [9,11,31]. The antioxidant response is further enhanced by the complementary action of different classes of bioactive compounds. Rutin, the predominant flavonoid, together with betalains and the highly unsaturated lipid fraction rich in squalene (2.4–12% of the oil), contributes to scavenging reactive oxygen species and protecting cellular components against oxidative damage. Nevertheless, the stability of these compounds may be partially compromised during industrial processing, particularly under severe extrusion or milling conditions, leading to reductions in antioxidant capacity depending on process intensity and formulation [18,30,44].

The high antioxidant potential of amaranth supports its growing interest as an ingredient for functional foods and nutraceutical formulations. By reducing oxidative stress, its bioactive compounds may contribute to the prevention of chronic non-communicable diseases, including cardiovascular disorders and type 2 diabetes, although further clinical evidence is required to fully establish these health benefits [4,18,28]. Among cultivated *Amaranthus* species, amaranth consistently demonstrates one of the highest antioxidant capacities reported in the literature. Studies comparing *Amaranthus caudatus* with *Amaranthus cruentus* indicate markedly higher antioxidant activity, while the thermal stability of lipophilic compounds such as squalene allows much of their functionality to be retained after high-temperature processes, including popping [12,30,39]. These characteristics reinforce the potential of amaranth as a valuable functional ingredient for the development of health-promoting food products.

### 5.2. Anti-Inflammatory Properties

Amaranth has attracted increasing attention because of its anti-inflammatory potential, which is primarily attributed to the combined action of polyphenols, bioactive peptides, and unsaturated lipids [10]. Experimental studies have shown that protein hydrolysates derived from amaranth reduce the production of pro-inflammatory mediators and modulate key cytokines involved in inflammatory responses, highlighting their potential to alleviate intestinal inflammation and improve gut health [23,24].

Current evidence indicates that these effects are largely mediated by low-molecular-weight peptides released during germination, enzymatic hydrolysis, or gastrointestinal digestion. These peptides modulate the NLRP3 inflammasome and suppress signaling pathways regulated by nuclear factor kappa-B (NF-κB) and mitogen-activated protein kinases (MAPKs), thereby reducing the expression of inflammatory mediators induced by stimuli such as lipopolysaccharide. Collectively, these mechanisms contribute to preserving epithelial barrier integrity and limiting chronic inflammatory responses [24,36,45].

Despite these promising findings, the biological activity of amaranth-derived peptides may vary under physiological conditions. Their effectiveness is influenced by gastrointestinal digestion, interactions with the gut microbiota, and the surrounding food matrix, factors that may partially explain the differences observed between in vitro and in vivo studies and highlight the need for additional clinical investigations [24,36,45].

The anti-inflammatory properties of amaranth support its application in the development of functional foods and nutraceutical products aimed at reducing chronic low-grade inflammation. Such properties are particularly relevant for metabolic disorders associated with obesity and type 2 diabetes, where persistent inflammation plays a central role in disease progression [24,31]. Moreover, because amaranth is naturally gluten-free, it represents an attractive dietary ingredient for individuals with celiac disease, avoiding the immune activation and intestinal inflammation triggered by gluten-derived peptides [23,44].

Similar anti-inflammatory mechanisms have been described in other Amaranthus species, such as *Amaranthus hypochondriacus* L., and in pseudocereals including quinoa. Nevertheless, amaranth appears to distinguish itself through the production of multifunctional peptides that simultaneously exhibit antioxidant and anti-inflammatory activities while maintaining favorable bioavailability [7,31,36]. These characteristics reinforce its potential as a high-value plant-based ingredient for the formulation of health-promoting foods and nutraceutical products.

### 5.3. Cardiometabolic Effects

Growing evidence indicates that amaranth consumption may contribute to improvements in several cardiometabolic risk factors. In animal models, experimental studies have reported reductions of up to 20% in total cholesterol and 36% in triglyceride concentrations, together with decreases in systolic blood pressure from 200 to 220 to 140–170 mmHg in hypertensive models [15,36]. These findings provide mechanistic support for potential cardiometabolic effects but cannot be directly extrapolated to humans. In contrast, limited human intervention evidence involving supplementation with 18 mL·day−1 of amaranth oil has been associated with improved lipid profiles, characterized by increased HDL cholesterol and reduced LDL and VLDL concentrations in individuals with coronary artery disease and hypertension [4,17,30].

These cardiometabolic effects appear to result from the complementary action of multiple bioactive constituents. Squalene and phytosterols contribute to cholesterol homeostasis by modulating HMG-CoA reductase activity, thereby reducing endogenous cholesterol synthesis. At the same time, bioactive peptides released during digestion inhibit angiotensin-converting enzyme and dipeptidyl peptidase-4, mechanisms associated with improved vascular function, blood pressure regulation, and glucose metabolism [10,27,36]. Dietary fiber further enhances these effects by binding bile salts, increasing cholesterol excretion, and attenuating postprandial glucose absorption [10,44].

The combined action of these compounds highlights the potential of amaranth as a functional ingredient for dietary strategies targeting cardiometabolic health [35]. Its incorporation into functional foods may support the nutritional management of metabolic syndrome, cardiovascular disease, and type 2 diabetes, although the limited number of human studies, differences in dose and intervention duration, and variation in food matrix and bioavailability currently prevent definitive conclusions regarding the magnitude of these benefits in humans. Additional well-designed clinical trials are therefore needed to confirm the long-term efficacy suggested by experimental studies [10,45,46].

Comparable cholesterol-lowering effects have also been reported for other pseudocereals, including quinoa and buckwheat. Nevertheless, amaranth is distinguished by its exceptionally high squalene concentration, one of the highest reported among edible plant sources, together with a favorable profile of unsaturated fatty acids and bioactive peptides [12,25]. Although rapeseed oil has shown superior performance for specific markers of atherosclerosis in some studies, the available evidence suggests that the unique combination of lipid-soluble phytochemicals and antihypertensive peptides in amaranth provides a promising strategy for promoting cardiovascular health [4,23,36].

### 5.4. Prebiotic Potential

Amaranth has emerged as a promising source of prebiotic substrates owing to its high content of dietary fiber, resistant carbohydrates, and bioactive phytochemicals capable of modulating the intestinal microbiota [10,35]. Rather than acting as a probiotic itself, amaranth provides fermentable substrates that support the growth and metabolic activity of beneficial microorganisms, particularly species belonging to the genera *Lactobacillus* and *Bifidobacterium*.

The prebiotic effects of amaranth are mainly associated with microbial fermentation of its non-digestible carbohydrates and phenolic compounds in the colon. This process promotes the production of short-chain fatty acids (SCFAs), including acetate, propionate, and butyrate, metabolites that contribute to intestinal barrier integrity, immune regulation, and host metabolic health [10,15,35]. Compared with many refined cereal products, the higher fiber and phytochemical contents of amaranth provide a more favorable substrate for microbial fermentation, although direct comparative studies among cereals remain limited.

Evidence from traditional Andean fermented foods also supports the technological relevance of amaranth as a fermentation substrate. Fermented beverages such as *chicha de siete semillas* harbor complex microbial communities dominated by species including *Streptococcus macedonicus* and *Leuconostoc lactis*, demonstrating that amaranth can sustain the growth and metabolic activity of beneficial microorganisms during fermentation [47].

These characteristics highlight the potential of amaranth for the development of symbiotic foods, in which prebiotic substrates are combined with probiotic microorganisms to promote gut health. In addition to stimulating beneficial microbiota, microbial fermentation contributes to the degradation of phytic acid and other antinutritional factors, thereby improving mineral bioavailability and enhancing the nutritional quality of the final product [15,30,31]. Despite these promising findings, further research is required to establish the optimal formulations, probiotic strains, dosages, and product stability necessary to ensure consistent health benefits under different dietary conditions and in human intervention studies.

## 6. Amaranth Processing Technologies

Amaranth is a highly versatile raw material whose nutritional, functional, and technological properties are strongly influenced by processing conditions [3,4,48]. Its industrial use involves both conventional technologies, such as milling, roasting, popping, flaking, and extrusion, and biotechnological approaches, including germination, fermentation, enzymatic hydrolysis, encapsulation, and component isolation. These processes improve palatability [9,17], digestibility, bioactive compound availability, and techno-functional performance, while also influencing texture, sensory quality, and industrial applicability [1,11,43].

### 6.1. Milling

Milling is the primary operation used to transform amaranth grains into flour by reducing particle size and improving ingredient uniformity [25,49]. Unlike wheat, which separates the endosperm from the germ and pericarp, amaranth is commonly processed as a whole grain through cleaning, moisture conditioning, milling (commonly in hammer or disk mills), and sieving to ensure homogeneity. This approach preserves dietary fiber, minerals, and bioactive compounds, but also creates technological challenges due to the extremely small grain size and the tendency to generate fine particles and dust during milling [8,48,49].

The particle size distribution of amaranth flour directly affects its hydration properties, dough rheology, and performance in food formulations. Although amaranth can produce finer flours than wheat or quinoa under similar conditions, excessive milling may broaden particle size distribution and alter water absorption, pasting behavior, and dough handling properties. Therefore, pretreatment and milling intensity must be optimized according to the intended application [25,31,49].

### 6.2. Roasting

Roasting is a dry-heat process used to improve the flavor, aroma, texture, and palatability of amaranth grains [27]. During roasting, moisture is progressively removed, producing a crisp texture and promoting chemical changes associated with flavor development and bioactive compound release. Compared with raw grains, roasted amaranth may exhibit increased total phenolic content, with values rising from approximately 14.72 mg GAE·100 g^−1^ to 662.78 mg GAE·100 g^−1^, exceeding those reported for boiled grains [4,37,50].

This increase in phenolic compounds, flavonoids, and phenolic acids contributes to improved antioxidant capacity, as measured by ABTS and FRAP assays [22,30,37]. However, roasting intensity must be carefully controlled. Moderate temperatures, around 145 °C, may enhance antioxidant concentration and improve rheological properties, whereas excessive heating above 190 °C can promote thermal oxidation and degradation of phenolics and flavonoids [22,28]. High temperatures may also reduce protein solubility and available lysine through Maillard reactions and protein cross-linking, with losses ranging from 7.3 to 11.9% [17,35]. Thus, roasting can modify the sensory and nutraceutical properties of amaranth, with the extent of these changes depending on the applied heat intensity and processing conditions [22,35].

### 6.3. Popping

**Figure 2:**
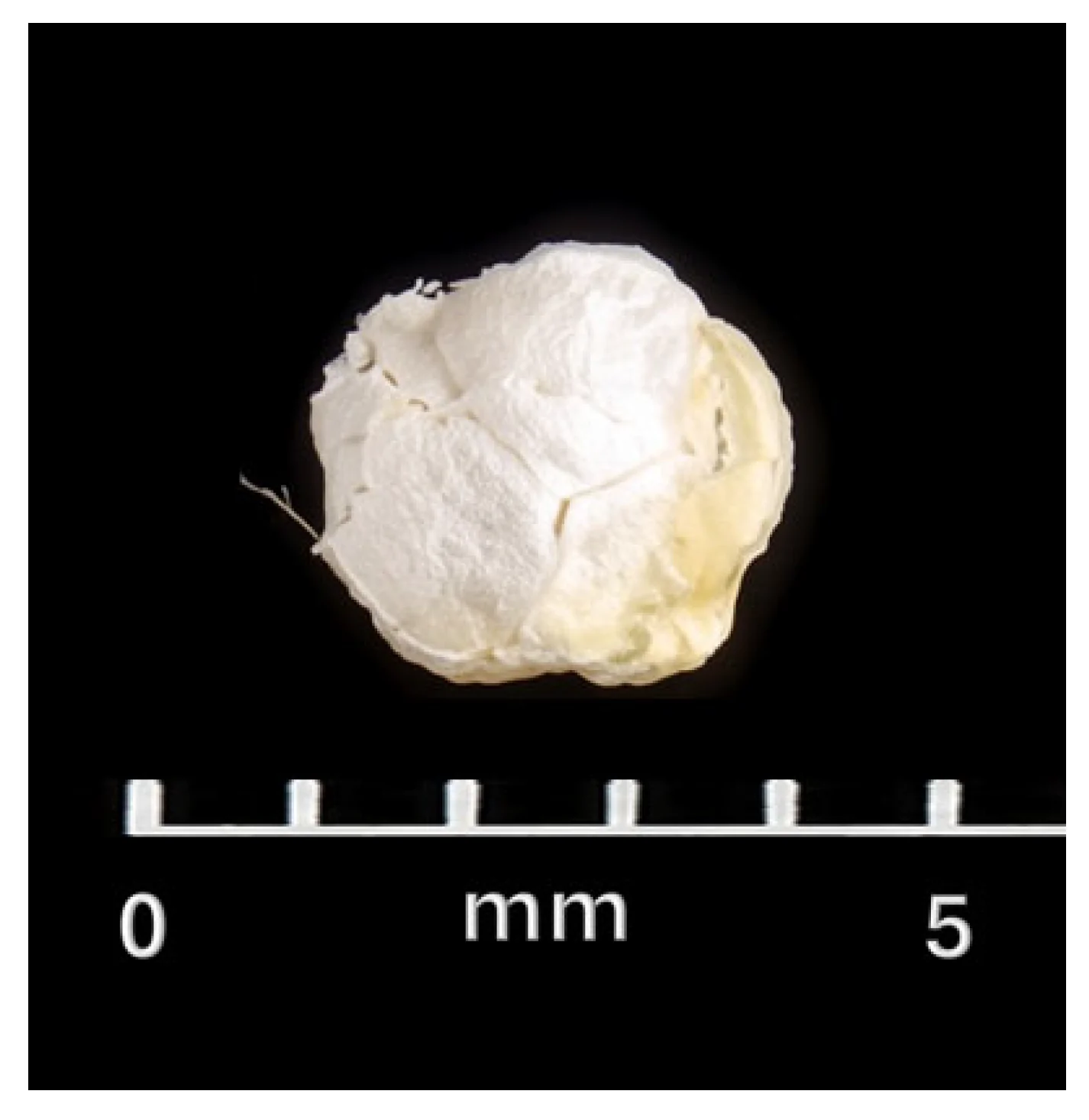
Close-up view of a popped amaranth seed showing its physical appearance and size, with a reference scale in millimeters.

Popping, or puffing, is a short-time dry-heat process in which amaranth seeds are exposed to high temperatures, typically between 160 and 200 °C for 15–30 s. The rapid vaporization of internal moisture causes the grain to expand, generating a porous and crunchy structure. Figure 2 shows a close-up macro photograph of a single popped amaranth grain, highlighting its expanded structure and providing a millimeter-scale reference for its size [9,16,17].

Puffed amaranth has enhanced sweetness, improved texture, and greater consumer acceptability compared with raw grains. It can also produce flours with peak and final viscosities closer to those of wheat flour than those of unprocessed whole-grain amaranth [27,48]. Nutritionally, popping may increase the relative concentration of proteins and lipids and improve starch digestibility. It also reduces antinutritional factors such as phytates, thereby improving mineral bioavailability [15,25,48].

However, uncontrolled popping conditions may negatively affect protein quality, especially available lysine, with losses of up to 11.9% reported under certain processing conditions [15,17,48]. Therefore, processing temperature, residence time, grain moisture, and varietal characteristics must be optimized to balance expansion, sensory quality, digestibility, and nutritional preservation.

### 6.4. Flaking

Flaking is a mechanical pre-cooking process that transforms whole amaranth grains into flattened flakes using a double-drum or roller system. The process generally requires prior grain moistening to approximately 18% moisture, followed by passage through narrow roller gaps under controlled temperature and rotation conditions [17].

Unlike popping, which produces a light and expanded structure through sudden vaporization, flaking generates a denser and flatter matrix. These structural differences directly affect product texture and sensory perception. Amaranth flakes used in products such as energy bars tend to increase stickiness while reducing hardness, stiffness, and crunchiness compared with expanded grains [17,48]. Therefore, flaking is particularly suitable for applications where a compact structure, moisture retention, and cohesive texture are desired.

### 6.5. Extrusion

Extrusion is a high-temperature, short-time continuous process that combines mechanical and thermal energy to gelatinize starch, denature proteins, and reorganize food matrices into expanded and textured products [9,27,30]. The process involves mixing, cooking, kneading, shearing, and shaping under pressure using single- or twin-screw extruders [9].

Extrusion can improve the nutritional and functional properties of amaranth by increasing starch and protein digestibility, inactivating antinutritional factors, and promoting the release of phenolic compounds bound to the cell wall. In amaranth, extrusion at 160 °C has been associated with a 20.32% increase in total phenolic content [9]. Protein digestibility may also reach values up to 96.3% in products formulated for infant nutrition using varieties such as Centenario and Oscar Blanco [2,7].

Despite these advantages, amaranth presents technological challenges during extrusion. Its high protein and fiber contents can interfere with steam bubble formation at the die exit, limiting expansion and resulting in denser products compared with corn-based snacks [9,51]. Moreover, excessive temperatures, particularly above 190 °C, may degrade essential amino acids and promote polyphenol oxidation [9,30]. Therefore, moisture content, barrel temperature, screw speed, die geometry, and formulation must be optimized to achieve desirable texture while preserving nutritional and functional quality.

### 6.6. Germination

Germination is a controlled bioprocess that reactivates seed metabolism through hydration, enzymatic hydrolysis, and reserve mobilization, initiating seedling development [1,11]. Compared with intense thermal treatments, germination is a low-cost and low-energy strategy that enhances nutritional quality while preserving heat-sensitive bioactive compounds [11,15,22].

This process substantially improves the functional value of amaranth. Germination can increase phenolic compounds by up to 284%, based on the increase in total phenolic content from 241 to 926 mg GAE·100 g^−1^ after 72 h of germination in a black-seeded ecotype [11]. This corresponds to a 3.84-fold increase in concentration, equivalent to a 284% increase relative to the raw grain. Germination can also enhance protein content, betalains, GABA, and antioxidant capacity [1,11]. It also activates endogenous phytases, reducing phytic acid and improving mineral bioavailability, while proteolytic enzymes contribute to increased protein digestibility [1,30,32].

The effectiveness of germination depends on time, temperature, humidity, ecotype, and hygienic control. Black-seeded amaranth ecotypes have been reported to accumulate higher levels of protein, ash, and total phenolic compounds after 72 h of germination, whereas pink ecotypes may contain higher betalain concentrations [11]. In a specific study, germination for approximately 63 h at 26 °C was reported as an effective condition for enhancing bioactive compound accumulation in amaranth [11]. However, this condition should be interpreted as study-specific rather than as a universal optimum, since germination responses may vary according to genotype, ecotype, germination duration, temperature, and target response [6,15,22].

### 6.7. Fermentation

Fermentation is a traditional biotechnological process based on the metabolic activity of microorganisms, mainly lactic acid bacteria and yeasts, which transform grain components through enzymatic reactions [15,30,37]. In amaranth, fermentation can improve nutritional quality by increasing protein, lipid, and ash contents, while reducing antinutritional factors such as phytates, tannins, and oxalates [15,28,30].

Sourdough-type fermentation is particularly relevant because it enhances mineral bioavailability, promotes the release of soluble phenolic compounds, and may generate bioactive peptides with antihypertensive and antidiabetic potential [10,15,25]. Fermented amaranth also provides a suitable substrate for the development of symbiotic foods and traditional beverages, such as *chicha de siete semillas* [15,27,47].

Nevertheless, fermentation outcomes depend strongly on microbial strain, fermentation time, temperature, pH, and substrate composition. Poorly controlled conditions may limit antinutrient degradation or favor undesirable microbial growth. Therefore, standardization of fermentation protocols is essential for safe and reproducible industrial applications.

### 6.8. Encapsulation

Encapsulation is an emerging strategy used to protect bioactive compounds and improve their solubility, stability, and bioavailability. In amaranth, protein isolates and starches can act as biopolymeric matrices for encapsulating heat-sensitive or hydrophobic compounds [4,21,52].

This technology is particularly valuable because encapsulating matrices protect bioactive agents against oxidation, ingredient interactions, and degradation during processing and digestion. They may also enable controlled release in the intestinal phase, improving compound absorption and biological efficacy [21,52]. In amaranth, complex coacervation using ultrasound-treated proteins combined with polymers such as sodium carboxymethylcellulose has been explored for betanin microencapsulation.

Although promising, encapsulation using amaranth-derived matrices still requires further optimization. Future studies should standardize encapsulation efficiency, release kinetics, storage stability, sensory compatibility, and scalability for food applications [4,21].

### 6.9. Enzymatic Hydrolysis

Enzymatic hydrolysis is a controlled biotechnological process in which enzymes such as pepsin, pancreatin, or microbial proteases cleave proteins into smaller peptides with improved digestibility and biological activity [24,52]. In amaranth, hydrolysis is particularly relevant because its protein fractions are rich in albumins and globulins, which can release low-molecular-weight bioactive peptides. Amaranth hydrolysates have been associated with antioxidant, antihypertensive, antidiabetic, and potential anticancer activities. These effects are mainly attributed to peptides capable of inhibiting ACE and DPP-IV, as well as sequences such as lunasin, which may retain functionality after gastrointestinal digestion [24,30,45]. Hydrolysis can increase protein digestibility by more than 20% compared with intact grains and generate peptides with molecular weights between 0.16 and 3.11 kDa, favoring bioavailability [24,30].

These characteristics position amaranth hydrolysates as promising ingredients for functional foods and nutraceutical formulations. However, enzyme type, hydrolysis conditions, peptide stability, sensory impact, and bioactivity under in vivo conditions require further evaluation.

### 6.10. Isolation of Components

Component isolation comprises physicochemical processes used to separate, purify, and concentrate specific amaranth fractions, mainly protein isolates and starch [24]. Protein isolation is commonly performed using the pH-shift method, which involves alkaline extraction at pH 8.0–11.0 to solubilize albumins and globulins, followed by isoelectric precipitation at pH 4.0–5.5. Under acidic conditions, proteins precipitate and can be separated from fiber and soluble carbohydrates, allowing the production of high-purity protein fractions, often exceeding 80% protein [21,24].

Starch isolation, in turn, generally requires prolonged soaking in dilute sodium hydroxide or metabisulfite solutions to release starch granules from the surrounding protein matrix. This is followed by filtration through fine mesh screens and successive centrifugation steps to obtain a purified starch fraction [29,52].

Compared with salt- or solvent-based extraction methods, isoelectric precipitation generally provides amaranth protein isolates with higher yield and purity [53]. Starch recovery from amaranth may also be technically more efficient than from kañiwa, whose smaller granules tend to retain higher amounts of lipid and fiber impurities [29]. In comparison with quinoa, amaranth protein isolates may exhibit better solubility, as quinoa proteins are more susceptible to structural denaturation during chemical extraction [53].

Although component isolation enables the concentration of nutritionally valuable fractions and may reduce antinutritional factors such as tannins, the use of extreme alkaline and acidic conditions can induce protein conformational changes, affecting solubility, emulsifying capacity, gelation, and other techno-functional properties [36,53]. Nevertheless, amaranth protein isolates retain a balanced essential amino acid profile, particularly rich in lysine and methionine, supporting their use in functional foods, infant formulas, and nutritional supplements for populations with specific nutritional requirements [21,24,36].

Overall, processing technologies play a decisive role in determining the nutritional, functional, and technological performance of amaranth. Conventional methods such as milling, roasting, popping, flaking, and extrusion improve palatability, digestibility, and product applicability, whereas biotechnological approaches such as germination, fermentation, enzymatic hydrolysis, encapsulation, and component isolation enhance bioactive compound availability, mineral bioavailability, and functional performance. As summarized in Table 3, each process presents specific advantages and limitations, reinforcing the need to optimize processing parameters according to the target product, desired functionality, and industrial application.

## 7. Food Applications of Amaranth

The growing interest in amaranth as a functional ingredient has expanded its application across a wide variety of food products. Depending on the processing technology employed, amaranth can be incorporated as whole grains, raw or processed flours, germinated grains, puffed grains, protein isolates, starch, or edible oil, providing different technological and nutritional functionalities [11,17,23]. As summarized in Table 4, this versatility enables its incorporation into numerous food categories, including bakery products, pasta, breakfast cereals, extruded snacks, malted beverages, edible oils, fermented foods, gluten-free products, and high-quality infant formulations [2].

The incorporation of amaranth into food systems generally improves nutritional quality owing to its balanced amino acid profile, high dietary fiber content, and abundance of bioactive compounds. Replacing conventional cereal flours with amaranth can substantially increase lysine content while enhancing the levels of minerals, phenolic compounds, and other nutraceutical constituents [11,14,40]. Nevertheless, these nutritional advantages are frequently accompanied by technological challenges. Because amaranth is naturally gluten-free, high substitution levels may reduce dough elasticity, decrease loaf volume, and increase crumb firmness in baked products [14,21,54].

Several processing technologies have been successfully applied to overcome these limitations. Germination, extrusion, fermentation, and other bioprocesses improve mineral bioavailability, increase the concentration of compounds such as GABA and phenolics, and partially compensate for technological limitations by modifying starch and protein functionality [6,7,54]. These strategies contribute to improving both the nutritional value and the processing performance of amaranth-based foods without substantially compromising consumer acceptance.

Although products containing amaranth often exhibit a darker color and denser texture, mainly as a consequence of Maillard reactions and the absence of gluten, their characteristic nutty flavor is generally well accepted by consumers, resulting in favorable sensory acceptance across different food categories [14]. Collectively, these findings demonstrate that appropriate ingredient selection and process optimization are fundamental for maximizing the nutritional, technological, and sensory potential of amaranth in the development of value-added functional foods.

## 8. Technological and Industrial Challenges, Sustainability and Future Perspectives

Despite its outstanding nutritional and functional properties, the large-scale industrial utilization of amaranth remains constrained by several technological, processing, and regulatory challenges. One of the principal limitations is the absence of a gluten-forming protein network, which reduces gas retention during baking and compromises loaf volume, crumb structure, and texture when compared with conventional cereal-based products [28,54]. Likewise, the high protein and dietary fiber contents can interfere with steam bubble formation during extrusion, resulting in lower expansion and denser products than those obtained from refined cereals such as corn or rice [9,51].

The technological performance of amaranth-based foods is also influenced by structural interactions among starch, proteins, and dietary fiber. High substitution levels may produce darker products due to Maillard reactions and pigment degradation while simultaneously increasing firmness and modifying dough rheology through reduced protein network formation and altered water distribution within the matrix [14,42,55]. In addition, although amaranth possesses a favorable antioxidant profile, its unsaturated lipid fraction is susceptible to oxidation under severe thermal conditions, and excessive heat exposure may reduce the availability of essential amino acids such as lysine, emphasizing the importance of carefully optimizing processing conditions [8,9,17].

Beyond processing limitations, industrial scalability remains a major challenge. The extremely small grain size complicates mechanized sowing, harvesting, cleaning, and post-harvest handling, while the production of high-value ingredients, including protein isolates and purified bioactive peptides, still requires relatively expensive processing technologies [10,45]. Furthermore, differences in international regulations governing food fortification, labeling, and health claims, together with the risk of adulteration with other cultivated Amaranthus species, highlight the need for standardized quality certification systems, genetic authentication methods, and robust traceability programs to ensure commercial integrity and consumer confidence [3,12,39].

Despite these constraints, amaranth may contribute to more diversified and resilient food systems because of its adaptation to relatively marginal environments and its nutritional density. However, its contribution to sustainability should be evaluated according to specific production conditions, including agronomic practices, resource requirements, processing efficiency, and market development.

The industrial utilization of amaranth may also support circular economy strategies. Virtually all fractions of the grain, including whole flour, bran, starch, protein isolates, oil, and protein hydrolysates, can be transformed into high-value ingredients. Such comprehensive utilization may improve resource efficiency and reduce processing losses. Furthermore, integrating green extraction technologies, biorefinery strategies, and sustainable bioprocesses into the amaranth value chain may improve resource efficiency by maximizing the recovery of high-value bioactive compounds and reducing processing losses.

From a food security perspective, amaranth may contribute to dietary diversification because of its protein quality and micronutrient content; however, its actual contribution to food security depends on local production systems, affordability, accessibility, and consumer acceptance.

Looking ahead, advances in plant breeding, precision agriculture, food processing, artificial intelligence-assisted process optimization, encapsulation technologies, precision fermentation, and digital quality monitoring may expand the industrial applications of amaranth. These innovations, combined with increasing consumer demand for sustainable and health-promoting foods, could improve processing efficiency and product functionality. However, further validation at pilot and industrial scales, together with economic, environmental, and regulatory assessments, is required to determine the feasibility and sustainability of these approaches.

## 9. Conclusions

Amaranth (*Amaranthus caudatus* L.) is an Andean pseudocereal with a diverse nutritional composition and a range of functional and technological properties. The evidence compiled in this review indicates that amaranth provides proteins with a relatively favorable essential amino acid profile, dietary fiber, unsaturated lipids, minerals, and bioactive compounds, including squalene, polyphenols, tocopherols, phytosterols, and bioactive peptides. These components have been associated with antioxidant, anti-inflammatory, cardiometabolic, and prebiotic effects, although the strength of evidence varies according to the compound, experimental model, and processing conditions. These characteristics support the consideration of amaranth as an ingredient for the development of functional foods, nutraceuticals, and gluten-free products.

Among the processing technologies currently available, germination has been reported to modify the nutritional and functional properties of amaranth, including increases in phenolic compounds, GABA, protein digestibility, and mineral bioavailability, together with reductions in some antinutritional factors. Extrusion can modify digestibility and techno-functional properties and can be used to produce expanded products, whereas fermentation may enhance the availability of selected bioactive compounds and minerals and modify the functional properties of amaranth-based ingredients. In addition, emerging technologies such as enzymatic hydrolysis, encapsulation, and component isolation provide approaches for modifying the stability, bioaccessibility, and functionality of specific amaranth-derived compounds and ingredients. However, the magnitude and direction of these effects depend strongly on processing conditions, raw material characteristics, and the analytical methods employed.

Despite these advances, important challenges remain regarding processing optimization, sensory acceptability, industrial scalability, regulatory harmonization, and the clinical validation of the health effects associated with amaranth consumption. Future research should therefore prioritize the specific knowledge gaps identified in this review, including the characterization of underutilized ecotypes, investigation of genotype–environment interactions, standardization of processing and analytical methodologies, and well-designed human intervention studies. The application of innovative technologies, including precision processing, green extraction, biorefinery approaches, and digital process optimization, should be evaluated according to their effects on product quality, resource efficiency, scalability, and reproducibility rather than solely on their capacity to increase the concentration of individual bioactive compounds.

Future research should also directly address the specific knowledge gaps identified in this review. Particular attention is needed to characterize underutilized amaranth ecotypes, especially black- and pink-seeded materials, and to clarify genotype–environment interactions affecting nutritional and bioactive profiles. Greater standardization of processing conditions and analytical methodologies is also required to enable meaningful comparisons among studies. Moreover, although antioxidant, anti-inflammatory, and cardiometabolic effects have been reported, the available evidence remains predominantly based on in vitro and preclinical models; therefore, well-designed human clinical trials are essential to establish the relevance of these effects in humans and determine effective consumption levels. In addition, integrated studies linking processing conditions with molecular and structural changes, bioaccessibility, techno-functional properties, sensory quality, and biological responses are needed to support the development of reproducible and scalable amaranth-based food applications.

Beyond its nutritional relevance, amaranth may contribute to discussions on sustainable food systems, agrobiodiversity conservation, and resource-efficient food production. Its adaptability to marginal environments, reported tolerance to environmental stresses, and the potential valorization of different plant fractions may provide opportunities for diversification of production systems. Furthermore, integrating amaranth into diversified production chains could contribute to local value creation, conservation of Andean agrobiodiversity, and the development of food products with potentially lower environmental impacts, although these outcomes require evaluation through appropriate environmental, economic, and supply-chain assessments. Collectively, the available evidence supports further investigation of amaranth as a versatile raw material for food applications, while its contribution to nutrition, sustainability, and food security should be assessed in relation to specific production systems, processing technologies, and consumer contexts.

## Figures and Tables

**Table 1:** Nutritional comparison of amaranth and other grains and cereals (dry basis).

Component (%)	Amaranth	Quinoa	Kañiwa	Lupin	Buckwheat	Polished White Rice	Whole Yellow Corn	References
Protein	12.0–15.9	9.6–15.2	11.7–23.0	43.0–52.4	9.1–14.9	7.0	9.4	[2,7,21,23,25]
Lipids	2.2–13.7	4.0–15.2	4.5–8.9	18.0–21.4	2.0–3.6	1.0	4.7	[2,11,21,23,25]
Ash	2.0–3.8	2.3–5.5	2.6–3.7	2.7–2.8	1.8–2.8	0.4	1.2	[2,7,21,23,25]
Available Carbohydrates	55.5–65.7	59.4–70.8	52.4–69.5	14.5–31.0	66.3–72.3	80.3	74.3	[2,21,23,25]
Fiber	2.7–17.3	7.0–26.5	5.3–14.4	7.5–10.0	17.8	0.1	2.7	[2,7,21,23,25]

Note: Values are expressed as percentages (%) on a dry weight basis and are presented as ranges reported in the literature. Variability reflects differences in genotype (cultivar), geographical origin, environmental and agronomic conditions, maturity stage, postharvest handling, analytical methods, and processing conditions. References corresponding to each crop are provided in the last row of the table.

**Table 2:** Bioactive compounds in amaranth grains and amaranth-containing food products under different processing conditions.

Bioactive Compound	Amaranth Sample or Processing Condition	Reported Value	References
Total Phenolic Content (TPC)	Raw grain (*Amaranthus caudatus*, Black ecotype)	241 ± 1.84 mg GAE/100 g dw	[11,14]
	Germinated grain (*Amaranthus caudatus*, Black ecotype, 24 h)	655 ± 1.77 mg GAE/100 g dw	
	Germinated grain (*Amaranthus caudatus*, Black ecotype, 48 h)	869 ± 1.59 mg GAE/100 g dw	
	Germinated grain (*Amaranthus caudatus*, Black ecotype, 72 h)	926 ± 1.58 mg GAE/100 g dw	
	Raw grain (*Amaranthus caudatus*, Pink ecotype)	168 ± 1.81 mg GAE/100 g dw	
	Enriched Bread (PE) (wheat bread with 5% quinoa, 5% kiwicha, 5% oats, 5% wheat bran)	114.23 ± 1.81 mg GAE/100 g dw	
	Gluten-Free Bread with amaranth (PLGK) (46.3% amaranth flour, 53.7% potato starch)	101.06 ± 1.81 mg GAE/100 g dw	
	French Bread (PF) (100% wheat flour)	64.83 ± 0.48 mg GAE/100 g dw	
Total Betalains	Raw grain (*Amaranthus caudatus* Black ecotype)	0.257 ± 0.05 mg BT/100 g dw	[11]
	Germinated grain (*Amaranthus caudatus*, Black ecotype, 24 h)	0.710 ± 0.02 mg BT/100 g dw	
	Germinated grain (*Amaranthus caudatus*, Black ecotype, 48 h)	1.31 ± 0.12 mg BT/100 g dw	
	Germinated grain (*Amaranthus caudatus*, Black ecotype, 72 h)	1.57 ± 0.04 mg BT/100 g dw	

Note: Values reported for different processing treatments originate from independent studies and should not be interpreted as a single longitudinal dataset. Differences may reflect genotype, ecotype, geographical origin, processing conditions, extraction procedures, analytical methods, and reporting units. Direct comparisons should therefore be made only when analytical units, sample basis, extraction procedures, and processing conditions are comparable. TPC was determined using the Folin–Ciocalteu spectrophotometric method, whereas total betalains were determined using the Nilsson spectrophotometric method. GAE, gallic acid equivalents; dw, dry weight; BT, betalain equivalents.

**Table 3:** Technical and functional comparative analysis of processing technologies for amaranth.

Processing Technology	Processing Parameters	Structural/Physicochemical Changes	Main Outcomes	Technological Limitations	References
Milling	Hammer/disk mill; screen size: 0.20–0.50 mm.	Physical particle size reduction; increased surface-to-volume ratio; partial starch damage.	Improved hydration and flour uniformity; facilitates incorporation into bakery and pasta matrices.	Small seed size complicates efficient bran/germ separation; loss of minerals and phenolics if refined.	[8,25,31,48,49]
Roasting	110–150 °C; 3–30 min.	Thermal protein denaturation; disruption of phenolic-macromolecule complexes.	Enhanced nutty flavor and antioxidant extractability; increased total phenolic content (TPC).	Potential Maillard-induced reduction in available lysine; reduced protein solubility.	[4,17,22,27,35,37,50]
Popping	160–200 °C; 15–30 s; moisture: ~7%.	Vapor expansion; starch–protein network formation; perisperm disruption.	Instant texture; high in vitro protein digestibility (~96%); increased fiber bioaccessibility.	Significant lysine degradation (up to 11.9%); high volume-to-weight ratio increases transport costs.	[9,15,16,17,27,48]
Flaking	Double-drum roller; 120 °C; 11 s; 18% moisture.	Mechanical compression; partial starch gelatinization and dextrinization.	Improved adhesiveness and water binding; suitable for breakfast cereals and bars.	Energy-intensive pre-conditioning; specific moisture control required to prevent rancidity.	[17,48]
Extrusion	HTST; 100–180 °C (low) or 35–160 °C (high); 12–70% moisture.	Macromolecular unfolding; covalent bond cleavage; amylose–lipid complexation.	Maximized protein/starch digestibility; increased fiber solubility; versatile shapes and textures.	Thermal degradation of vitamins and pigments at >180 °C; high investment in equipment.	[7,9,27,30,51]
Germination	20–26 °C; 24–72 h; >80% RH.	Endogenous enzyme activation (phytases, amylases); cell wall weakening.	GABA synthesis (up to 699% increase); phytic acid reduction; mineral bioaccessibility gain.	Dry matter loss via respiration; risk of microbial growth; long processing cycle.	[1,6,11,15,22,30,32]

Note: HTST, high-temperature short-time; RH, relative humidity; TPC, total phenolic content; GABA, γ-aminobutyric acid. Processing parameters represent the ranges most frequently reported in the literature for amaranth (*Amaranthus caudatus* L.). Structural modifications and technological outcomes may vary depending on cultivar, grain characteristics, moisture content, equipment configuration, processing conditions, and product formulation.

**Table 4:** Applications of amaranth, impact on product properties, and technological barriers.

Products	Levels	Technological, Nutritional, and Sensory Properties	Technological Barriers	References
Baked Goods (sliced bread, French bread, and panettone)	Replace 5–40% of the wheat flour.	↑ Protein content (notably lysine). ↑ Dietary fiber content. ↑ Mineral content (iron, calcium). ↑ Total phenolic compounds. ↑ Shelf life by delaying starch retrogradation. ↑ Nutty flavor contribution. ↑ Sensory acceptability.	↓ Viscoelastic network formation (absence of gluten). ↓ Specific volume. ↑ Crumb firmness and density. ↑ Darker crumb coloration.	[4,14,15,20,21,25,27,40,54,55]
Gluten-Free Bakery Products	From 5 to 70% (using puffed grain).	↑ Protein quality compared to breads made with pure starches. ↑ Bioavailability of essential minerals. ↑ Nutritional value. ↑ Suitability for celiac diets (very low prolamin content).	↑ Water absorption capacity due to the high fiber content. ↓ Dough consistency and handling properties. ↓ Gas retention during fermentation. ↑ Dependence on hydrocolloids for structure stabilization.	[14,21,31,42,48]
Pasta and noodles (tagliatelle and fettuccine)	15–30% sprouted flour or up to 70% amaranth flour.	↑ GABA content (up to 4.5-fold increase after cooking). ↑ Antioxidant capacity (up to 8.7-fold increase, ORAC). ↑ Nutritional functionality through bioprocessing. ≈ Firmness and breaking strength comparable to the control in optimized formulations.	↓ Gluten network strength due to dilution. ↑ Loss of solids during cooking. ↓ Structural integrity if particle size is not controlled. ↑ Importance of particle size optimization.	[31,32]
Cookies and Pastries	25–60% substitution of wheat flour.	↑ Energy density. ↑ Essential amino acid content. ↑ Sensory acceptability (>70%). ↑ Color and flavor quality at substitution levels of around 25%.	↓ Spread ratio during baking. ↑ Excessive hardness in texture. ↑ Production costs compared to conventional cookies.	[30,54,56]
Snacks and energy/functional bars	Typically 11% of cracked or rolled grain.	↑ Protein content (~9.8 g/100 g). ↑ Caloric density. ↑ Squalene and phytosterol content. ↑ Porous, light, and crispy structure. ↑ Sensory appreciation/consumer acceptance.	↑ Changes in adhesiveness and hardness depending on the pre-cooking method (popping vs. flaking). ↑ Technological variability in bar texture due to processing conditions. ↓ Available lysine content due to intense heat treatment.	[17,35,51]
Beverages (plant-based milks, malted beverages, and beer)	70:30 blends (amaranth: tea) or 100% amaranth malt.	↑ Protein content (up to 2× higher than cow’s milk). ↑ Nutritional value. ↑ Prebiotic potential. ↑ Growth of probiotic bacteria such as Lactobacillus plantarum.	↑ Process complexity in amaranth malting. ↓ Viscosity stability during fermentation. ↑ Risk of viscosity loss due to enzymatic activity.	[4,21,22,23,25]

Note: ↑, increase or improvement; ↓, decrease or reduction; ≈, comparable to the control or no significant change. GABA, γ-aminobutyric acid; ORAC, oxygen radical absorbance capacity. Inclusion levels correspond to the ranges reported in the cited studies. Technological, nutritional, and sensory effects may vary depending on amaranth (*Amaranthus caudatus* L.) variety, flour characteristics (native, sprouted, popped, or malted), particle size, processing conditions, formulation, and interactions with other ingredients.

## Data Availability

No new data were created or analyzed in this study. Data sharing is not applicable to this article.

## Abbreviations

The following abbreviations are used in this manuscript:

AAE	Essential Amino Acids
AANE	Non-Essential Amino Acids
ABTS	2,2′-Azino-bis (3-ethylbenzothiazoline-6-sulfonic acid)
DPPH	2,2-Diphenyl-1-picrylhydrazyl
ECA	Angiotensin-Converting Enzyme
DPP-IV	Dipeptidyl Peptidase IV
GABA	Gamma-Aminobutyric Acid
ORAC	Oxygen Radical Absorbance Capacity
FRAP	Ferric Reducing Antioxidant Power
TPC	Total Phenolic Content
GAE	Gallic Acid Equivalents
CAE	Caffeic Acid Equivalents
HTST	High-Temperature Short-Time
KPI	Amaranth Protein Isolate
HMG-CoA	3-Hydroxy-3-Methylglutaryl Coenzyme A
LDL	Low-Density Lipoprotein
HDL	High-Density Lipoprotein
VLDL	Very Low-Density Lipoprotein
SCFA	Short-Chain Fatty Acids
NF-κB	Nuclear Factor Kappa B
MAPK	Mitogen-Activated Protein Kinase
GI	Glycemic Index
BV	Biological Value
PER	Protein Efficiency Ratio

## References

[1] De-La-Cruz-Yoshiura S., Vidaurre-Ruiz J., Alcázar-Alay S., Encina-Zelada C.R., Cabezas D.M., Correa M.J. (2023). Sprouted Andean Grains: An Alternative for the Development of Nutritious and Functional Products. Food Rev. Int..

[2] Repo-Carrasco-Valencia R., Basilio-Atencio J., Luna-Mercado G.I., Pilco-Quesada S., Vidaurre-Ruiz J. (2021). Andean Ancient Grains: Nutritional Value and Novel Uses. Biol. Life Sci. Forum.

[3] Ramos E., Coles P.S., Chavez M., Hazen B. (2022). Measuring Agri-Food Supply Chain Performance: Insights from the Peruvian kiwicha Industry. Benchmarking.

[4] Kaur S., Ghosh A., J. S., Kaur G., Singh G., Pandey P. (2025). Pseudocereals for Modern Diets: Multifunctional Grains with Superior Bioactive Properties, Nutraceutical Potential, and Diverse Industrial Applications. Food Chem. Adv..

[5] Vásquez-Ocmín P.G., Marti G., Gadea A., Cabanac G., Vásquez-Briones J.A., Casavilca-Zambrano S. (2023). Metabotyping of Andean Pseudocereals and Characterization of Emerging Mycotoxins. Food Chem..

[6] Paucar-Menacho L.M., Símpalo-López W.D., Castillo-Martínez W.E., Esquivel-Paredes L.J., Martínez-Villaluenga C., Schmiele M. (2024). Optimization of Rheological Properties of Bread Dough with Substitution of Wheat Flour for Whole Grain Flours from Germinated Andean Pseudocereals. Cienc. Rural.

[7] Osei E.D., Afedzi A.E.K., Amotoe-Bondzie A., Ivanišová E., Encina-Zelada C.R., Czaplicki S. (2025). Nutritional and Bioactive Characterization of *Amaranthaceae* Seeds from Peru, Slovakia, and Poland: A Comparative Study. Food Sci. Nutr..

[8] Dávalos J.Z., Tirado A., Romero V., Cisneros G., Gamarra F. (2023). Structural, Thermal and Energetic Properties of Andean Pseudocereal Flours with High Nutritional Values. J. Therm. Anal. Calorim..

[9] Zapana F., Vidaurre-Ruiz J., Linares-García L., Repo-Carrasco-Valencia R. (2025). Exploring the Future of Extrusion with Andean Grains: Macromolecular Changes, Innovations, Future Trends and Food Security. Plant Foods Hum. Nutr..

[10] Segura-Jiménez M.E., Jacobo-Velázquez D.A. (2025). *Amaranthus* spp.: A Multifunctional Crop at the Nexus of Nutrition, Health, and Sustainable Innovation. J. Agric. Food Res..

[11] Sarmiento-Aguilar W., Pachari-Vera E. (2022). Impact of germination on the bioactive compounds of two ecotypes of black and pink kiwicha grains (*Amaranthus caudatus* L.). Rev. Chil. Nutr..

[12] Terzieva S., Baycheva S., Tzanova M., Ivanova T., Dimitrova D., Grozeva N.H. (2026). Multifunctional Edible Amaranths: A Review of Nutritional Benefits, Anti-Nutritional Factors, and Potential in Sustainable Food Systems. Foods.

[13] Comettant-Rabanal R., Fuentes-Guevara D.Y., Delgado-Soriano V., Encina-Zelada C.R., Chávez-Llerena R.T., Rimari-Barzola R. (2025). Andean Lupin and Pecan Cakes as Sustainable Ingredients for Breakfast Cereals: A By-Product Approach to Functional Food Development. Appl. Food Res..

[14] Sotelo-Méndez A., García-Ramón D.F., Norabuena E., Naupay-Vega M., Sumarriva L., Álvarez H. (2025). Perfil Nutricional, Valor Biológico y Aceptabilidad General de Panes: Evaluación Comparativa de Formulaciones Tradicionales, Enriquecidas y Sin Gluten. Nutr. Clín. Diet. Hosp..

[15] Reddy M.V.K., Dubey P.K., Mishra A.A., Ahada-Sabeel S. (2024). Potential Processing Techniques for Safe Utilisation of Pseudocereals in the Food System. J. Food Compos. Anal..

[16] Miranda L., Huillca J., Marques-Pérez I. (2024). El Cultivo Reciente de Kiwicha (*Amaranthus caudatus* L.) en el Perú: Expansión de Producción y Comercialización. Agroalimentaria.

[17] Burgos V.E., Castillo V.C.D. (2021). Utilización de Amaranth Precocida (*Amaranthus caudatus*) para el Desarrollo de Barras Funcionales. Rev. Chil. Nutr..

[18] Netshimbupfe M.H., Berner J., Van Der Kooy F., Oladimeji O., Gouws C. (2023). The Importance and Use of *Amaranthus* for Crop Diversification in the SADC Region. S. Afr. J. Bot..

[19] Quispe-Sanchez L., Mestanza M., Goñas M., Gill E.R.A., Oliva-Cruz M., Chavez S.G. (2022). Physical, Functional and Sensory Properties of Bitter Chocolates with Incorporation of High Nutritional Value Flours. Front. Nutr..

[20] Chamorro-Gómez R.E., Abad-Villar D., Natividad-Bardales Á.D., Estacio-Laguna R., Ríos-García G., Muñoz-Garay S.G. (2023). Evaluación de las características fisicoquímicas y sensoriales del pan de molde enriquecido con *Amaranth* (*Amaranthus caudatus* L.) y cañihua (*Chenopodium pallidicaule* Aellen). Rev. Colomb. Investig. Agroind..

[21] Vidaurre-Ruiz J., Bender D., Schönlechner R. (2023). Exploiting Pseudocereals as Novel High-Protein Grains. J. Cereal Sci..

[22] Paucar-Menacho L.M., Salvador-Reyes R., Castillo-Martínez W.E., Símpalo-López W.D., Verona-Ruiz A., Lavado-Cruz A. (2022). Use of Andean Pseudocereals in Beer Production. Sci. Agropecu..

[23] Guevara D., Viteri C., Robayo V., Hidalgo K., Arteaga C. (2025). The Potential of Andean Foods in the Dietary Management of Celiac Disease: Nutritional Benefits and Practical Applications. Salud Cienc. Tecnol..

[24] Paz S.M.L., Martinez-Lopez A., Villanueva-Lazo A., Pedroche J., Millan F., Millan-Linares M.C. (2021). Identification and Characterization of Novel Antioxidant Protein Hydrolysates from Amaranth (*Amaranthus caudatus* L.). Antioxidants.

[25] Nandan A., Koirala P., Dutt Tripathi A., Vikranta U., Shah K., Gupta A.J. (2024). Nutritional and Functional Perspectives of Pseudocereals. Food Chem..

[26] Kanbar A., Beisel J., Gutierrez M.T., Graeff-Hönninger S., Nick P. (2023). Peruvian Amaranth (kiwicha) Accumulates Higher Levels of the Unsaturated Linoleic Acid. Int. J. Mol. Sci..

[27] Kour R., Jan T., Ahmed N., Sheikh M.A., Ubaid M., Yadav N., Kumar K., Sheikh I., Chauhan P., Shreaz S. (2025). A Comprehensive Exploration of Compositional Characteristics, Bioactive Compounds, Anti-Nutritional Factors, and Food Applications of Amaranths. J. Appl. Biol. Biotechnol..

[28] Lopez-Martinez J.M., Ahmad I. (2024). Amaranth Seeds: A Promising Functional Ingredient for Gastronomy—A Review. Sarhad J. Agric..

[29] Mérida-López J., Rojas C.C., Bergenståhl B., Purhagen J. (2024). Functional Properties of Starch Cultivars of Two Andean Grains Grown in Bolivia: Amaranth (*Amaranthus caudatus*) and Cañihua (*Chenopodium pallidicaule*). Heliyon.

[30] Jan N., Hussain S.Z., Naseer B., Bhat T.A. (2023). Amaranth and Quinoa as Potential Nutraceuticals: A Review of Anti-Nutritional Factors, Health Benefits and Their Applications in Food, Medicinal and Cosmetic Sectors. Food Chem. X.

[31] Paucar-Menacho L.M., Vásquez-Guzmán J.C., Símpalo-López W.D., Castillo-Martínez W.E., Martínez-Villaluenga C. (2023). Enhancing Nutritional Profile of Pasta: The Impact of Sprouted Pseudocereals and Cushuro on Digestibility and Health Potential. Foods.

[32] Paucar-Menacho L.M., Schmiele M., Vásquez-Guzmán J.C., Rodrigues S.M., Símpalo-López W.D., Castillo-Martínez W.E. (2024). Smart Pasta Design: Tailoring Formulations for Technological Excellence with Sprouted Quinoa and Amaranth Grains. Foods.

[33] Mérida-López J., Pérez S.J., Bergenståhl B., Purhagen J., Rojas C.C. (2023). Nutritional Composition of Six Amaranth (*Amaranthus caudatus*) Andean Varieties. Crops.

[34] Vidal Torres E., Valencia E., Simsek S., Ramírez A.M.L. (2024). Amaranth: Multipurpose Agroindustrial Crop. Agronomy.

[35] Singh N., Samarth R.M., Vashishth A., Pareek A. (2024). *Amaranthus* as a Potential Dietary Supplement in Sports Nutrition. CyTA J. Food.

[36] Rivero Meza S.L., Hirsch Ramos A., Cañizares L., Raphaelli C.D.O., Bueno Peres B., Gaioso C.A. (2023). A Review on Amaranth Protein: Composition, Digestibility, Health Benefits and Food Industry Utilisation. Int. J. Food Sci. Technol..

[37] Zhu F. (2025). Phenolic Compounds in Quinoa and Amaranth Grains: Composition, Biological Properties and Processing Effects. Food Chem..

[38] Paucar-Menacho L., Símpalo-López W., Castillo-Martínez W., Esquivel-Paredes L., Martínez-Villaluenga C. (2022). Improving Nutritional and Health Benefits of Biscuits by Optimizing Formulations Based on Sprouted Pseudocereal Grains. Foods.

[39] Kanbar A., Beisel J., Wetters S., Gutierrez M.T., Graeff-Hönninger S., Nick P. (2022). A Rapid, Simple, and Reliable Assay to Authenticate Peruvian Amaranth (*A. caudatus*) for Food Applications. Eur. Food Res. Technol..

[40] Miranda-Ramos K.C., Haros C.M. (2023). Influence of Pre-Baking and Frozen Storage on the Technological and Nutritional Quality of a Multi-Seed Bread. LWT.

[41] Yilma M.T., Eifa A., Belayneh M., Orsango A.Z. (2025). Effect of amaranth-containing dietary intervention in improving hemoglobin concentration: A systematic review and meta-analysis. Public Health Rev..

[42] Vidaurre-Ruiz J., Salas-Valerio F., Schoenlechner R., Repo-Carrasco-Valencia R. (2021). Rheological and textural properties of gluten-free doughs made from Andean grains. Int. J. Food Sci. Technol..

[43] Paucar-Menacho L., Símpalo-López W., Castillo-Martínez W., Esquivel-Paredes L., Martínez-Villaluenga C. (2022). Reformulating bread using sprouted pseudo-cereal grains to enhance its nutritional value and sensorial attributes. Foods.

[44] Khurana R., Kaur A., Gupta P. (2023). A systematic review and meta-analysis of a traditional pseudocereal—*Amaranthus cruentus*. J. Food Chem. Nanotechnol..

[45] Ojha P., Xia T., Zhang L., Sun Q., Chen S., Chitrakar B., Jielin L., Adhikari B.M., Karki R., Yaxin L. (2025). Advancements in in vitro simulated digestion-derived peptides for type 2 diabetes mellitus management: From therapeutic challenges to dietary opportunities. Food Chem. X.

[46] Mendoza Ocampo E.S., Gutiérrez Durán M.P., Grados Torrez R.E., Gonzáles Dávalos E.L. (2025). Effect of a natural nutraceutical product based on *Amaranthus caudatus*, *Chenopodium quinoa*, and *Lupinus mutabilis* on serum levels of sodium, potassium, and uric acid as indicators for achieving homeostasis. Gac. Med. Boliv..

[47] Rebaza-Cardenas T., Montes-Villanueva N.D., Fernández M., Delgado S., Ruas-Madiedo P. (2023). Microbiological and physicochemical characteristics of the Peruvian fermented beverage “Chicha de Siete Semillas”. Int. J. Food Microbiol..

[48] Chaquilla-Quilca G., Islas-Rubio A.R., Vásquez-Lara F., Salcedo-Sucasaca L., Silva-Paz R.J., Luna-Valdez J.G. (2024). Chemical, physical, and sensory properties of bread with popped amaranth flour. Pol. J. Food Nutr. Sci..

[49] Jamanca-Gonzales N.C., Ocrospoma-Dueñas R.W., Eguilas-Caushi Y.M., Padilla-Fabian R.A., Silva-Paz R.J. (2024). Techno-functional properties and rheological behavior of quinoa, amaranth, wheat flours and their mixtures. Molecules.

[50] Hu F., Xiong X., Ogawa Y. (2024). Effect of grain structure and cooking method on starch digestibility and antioxidant activity of amaranth (*Amaranthus caudatus* L.) grains during simulated gastrointestinal digestion. Food Chem. Adv..

[51] Barraza-Jáuregui G., Valderrama-Amasifuen F., Arteaga H., Flores A., Obregón J. Snacks a base de maíz morado, quinua y amaranth: Características físicas y sensoriales. Proceedings of the 19th LACCEI International Multi-Conference for Engineering, Education and Technology.

[52] Perez-Rea D., Antezana-Gomez R. (2024). The functionality of pseudocereal starches. Starch in Food.

[53] Yan X., Zhu F. (2025). Structural, physicochemical and functional properties of quinoa, buckwheat and amaranth protein isolates: A comparative study. Food Chem..

[54] Feitosa B.F., Barros J.H.T., Feitoza J.V.F. (2024). Pseudocereals—A bibliometric analysis and literature review on the potential for manufacturing flours, bakery products and milk analogues. NFS J..

[55] García-Ramón F., Sotelo-Méndez A., Alvarez-Chancasanampa H., Norabuena E., Sumarriva L., Yachi K. (2023). Influence of Peruvian Andean grain flours on the nutritional, rheological, physical, and sensory properties of sliced bread. Front. Sustain. Food Syst..

[56] Fernandes Almeida R., Borges L.A., Anacleto D., De Souza M.S.N., Da Silva L.B.M., Monroy Y.M. (2025). Gluten-free cookies: A comprehensive review of substitutes for wheat flour. Food Humanit..

